# Research progress of vascularization strategies of tissue-engineered bone

**DOI:** 10.3389/fbioe.2023.1291969

**Published:** 2024-01-19

**Authors:** Nanning Lv, Zhangzhe Zhou, Mingzhuang Hou, Lihui Hong, Hongye Li, Zhonglai Qian, Xuzhu Gao, Mingming Liu

**Affiliations:** ^1^ Department of Orthopedic Surgery, The First Affiliated Hospital of Soochow University, Suzhou, Jiangsu, China; ^2^ Department of Orthopedic Surgery, The Second People’s Hospital of Lianyungang Affiliated to Kangda College of Nanjing Medical University, Lianyungang, Jiangsu, China; ^3^ Department of Orthopedic Surgery, The Affiliated Lianyungang Clinical College of Xuzhou Medical University, Lianyungang, Jiangsu, China; ^4^ Department of Orthopedic Surgery, The Affiliated Lianyungang Clinical College of Jiangsu University, Lianyungang, Jiangsu, China

**Keywords:** osteogenesis, bone tissue engineering, bone defect, biological materials, angiogenesis

## Abstract

The bone defect caused by fracture, bone tumor, infection, and other causes is not only a problematic point in clinical treatment but also one of the hot issues in current research. The development of bone tissue engineering provides a new way to repair bone defects. Many animal experimental and rising clinical application studies have shown their excellent application prospects. The construction of rapid vascularization of tissue-engineered bone is the main bottleneck and critical factor in repairing bone defects. The rapid establishment of vascular networks early after biomaterial implantation can provide sufficient nutrients and transport metabolites. If the slow formation of the local vascular network results in a lack of blood supply, the osteogenesis process will be delayed or even unable to form new bone. The researchers modified the scaffold material by changing the physical and chemical properties of the scaffold material, loading the growth factor sustained release system, and combining it with trace elements so that it can promote early angiogenesis in the process of induced bone regeneration, which is beneficial to the whole process of bone regeneration. This article reviews the local vascular microenvironment in the process of bone defect repair and the current methods of improving scaffold materials and promoting vascularization.

## 1 Introduction

The increasing incidence of bone defects due to trauma, inflammation, tumor resection, pathological fractures, impaired blood supply, and congenital deformities with increasing population aging has dramatically increased demand for bone substitute materials (Yin et al., 2018; Chen et al., 2021). Although autologous bone transplantation is the gold standard for clinical treatment of bone defects, it has serious disadvantages, such as limited sources, many complications, and postoperative pain in the donor site (Cheng et al., 2020). In addition, allogeneic bone transplantation is also an effective way to treat bone defects. It can avoid the shortcomings of lack of bone source and secondary damage to the material obtained, but it still faces problems such as immune rejection and disease transmission (Juhl et al., 2019). Therefore, designing and constructing safe and efficient bone repair biomaterials for treating bone defects has become one of the hotspots in bone regenerative medicine.

In bone repair, bone regeneration and angiogenesis are closely linked. As an indispensable source of nutrients for bone tissue, blood vessels transport minerals and growth factors to promote calcium deposition in bones and release paracrine signals to regulate the growth, differentiation, and regeneration of different types of cells (Filipowska et al., 2017; Zheng et al., 2021). Bone repair materials with good biological activity should promote angiogenesis in and around the bone defect and promote the osteogenic differentiation of bone precursor cells, ultimately beneficial to bone defect healing and new bone formation. Although significant progress has been made in the application of tissue-engineered bone in treating bone defects, it still faces slow or absent vascularization obstacles. This makes it difficult for tissue-engineered bone to form an effective vascular network in a short period of time, resulting in the death of seed cells due to lack of nutrients, oxygen and excessive accumulation of metabolites (Quinlan et al., 2017).

For a long time, many researchers have been exploring the ideal artificial bone defect repair materials. The biomimetic porous structure functions as a natural matrix, supporting cell adhesion, growth, differentiation, and proliferation and positively impacting bone tissue integration and revascularization (Huang et al., 2021). Tissue growth and bone tissue reconstruction are promoted by adjusting the microstructure, including pore size, shape, porosity, and interconnected pore structure (Hayashi et al., 2020; Geng et al., 2021). Some researchers combine growth factors with biomaterials, trying to endow biomaterials with new functions. A number of growth factors promote angiogenesis, including vascular endothelial growth factor (VEGF) (Wu et al., 2020) and essential fibroblast growth factor (bFGF) (Zhao et al., 2020). Furthermore, bioactive ions can also be doped into bone repair materials to modify their properties. By ion doping modification, bone biomaterials’ original physical, chemical, and biological properties can be improved or enhanced (Lai et al., 2019; Luo et al., 2021). Doping an appropriate amount of metal ions enhances the original related properties of bone repair materials and provides necessary trace elements for the metabolism of bone tissue. Table 1 showed the strategies of scaffold functionalization techniques for vascular bone tissue engineering.

**TABLE 1:** Strategies of scaffold functionalization techniques for vascular bone tissue engineering.

Strategies	Type	Characteristic	Ref
Optimize physical and chemical properties	Surface morphology, porosity and pore size, pore structure	Recruit macrophage/monocyte and induce angiogenesis	Brydone et al. (2010), Chen et al. (2018), Liu et al. (2020), Huang et al. (2021), Jin et al. (2021)
		Reduce inflammation, promoting angiogenesis, and driving adhesion and osteogenesis of MSCs. Cell-material interaction promotion and MSC paracrine function modulation effects	
Loaded cytokine	VEGF, Deferoxamine, Angiopoietin 2	A sustained release	Yin et al. (2018), Kerouredan et al. (2019b), Wu et al. (2020)
		Mimicked the microenvironment of extracellular matrix	
		Promoted angiogenesis and improved repair of bone defects	
Loaded trace elements	Magnesium, Strontium, Cobalt, Copper	Improve surface bioactivity and lead to better osteogenesis and angiogenesis	Son et al. (2019), Lee et al. (2020), Ma et al. (2020), Sun et al. (2021a)
		Alter physicochemical properties of cells, enhance the osteogenesis and angiogenesis of bioceramic scaffolds	
		Accelerated host angiogenesis and immune responses	
		Improve mechanical strength and handling properties, improve both biological and physicochemical properties	

In recent years, tissue engineering technology has become a hot research topic due to the development of materials science and molecular biology. However, inadequate vascularization after biological scaffold implantation remains a significant hurdle in bone tissue engineering research. How to ensure that the scaffold material can be rapidly vascularized after transplantation into the body and how to establish a vascular network to provide sufficient nutrition for subsequent new bone formation is an urgent problem that needs to be solved. This article reviews the local microenvironment in the process of bone defect repair and the current progress of bone tissue engineering to improve and promote vascularization.

## 2 Mechanisms that link bone tissue vascularization and osteogenesis

There are four stages in the process of bone defect repair: hematoma formation, fibrous callus formation, and bone remodeling (Zhang et al., 2020). Many studies have indicated that the initiation of bone regeneration relies on the inflammatory reaction and the formation of new blood vessels. Vascularization and bone regeneration are two fundamental components in the process of bone healing (Lu et al., 2022). Bone is a highly vascularized hard tissue. Bone remodeling and metabolism depend on the interaction of the vascular-bone microenvironment built between osteoblasts, osteocytes, osteoclasts, osteogenic differentiation, blood vessels, and angiogenesis. Osteogenic differentiation and angiogenesis are coupled through particular forms and related pathways. Therefore, the close temporal and spatial connection between bone formation and angiogenesis is called “osteogenic differentiation-angiogenesis coupling” (Gallardo-Calero et al., 2019). Bone remodeling and metabolism depend on vascular interactions. Blood vessels maintain the callus' high metabolic demand for oxygen and nutrients and provide pathways for inflammatory cells, osteoblast/osteoclast progenitors, and fibroblasts to enter the defect (Sivan et al., 2019). Factors that promote angiogenesis include VEGF, hypoxia-inducible factor-1α (HIF-1α), bone morphogenetic protein (BMP), FGF, transforming growth factor (TGF), and platelet-derived growth factor (PDGF-BB) are involved in initiating angiogenesis during bone formation and regulating bone formation (Peng et al., 2020).

### 2.1 H-type blood vessels promote osteogenic differentiation

**FIGURE 1:**
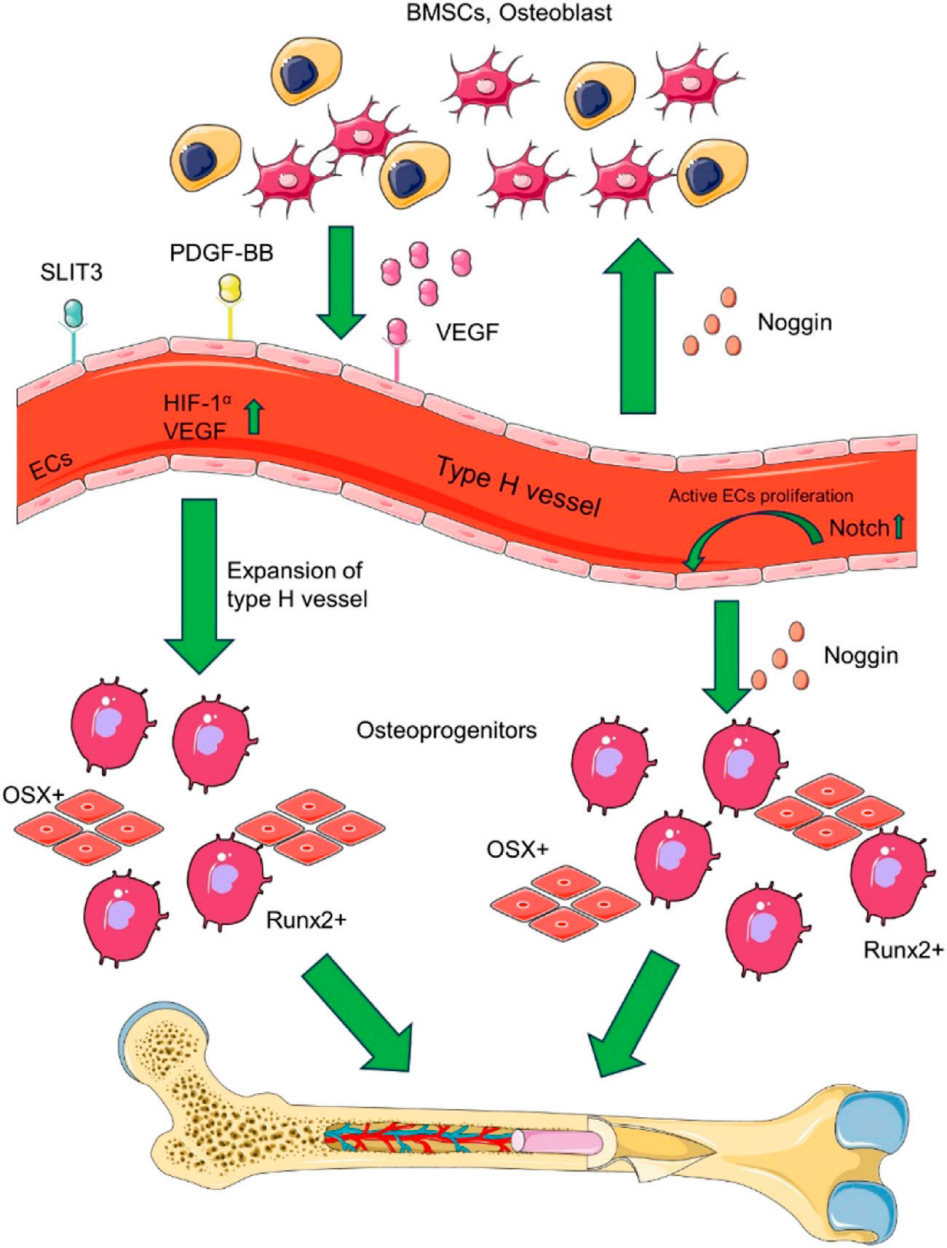
The coupling of type H vessel formation and osteogenesis in the bone marrow microenvironment involves multiple signalling factors. Various cell lineages, such as osteoblast lineage cells, vascular endothelial cells (ECs), and osteoclasts, contribute to the formation of type H vessels by secreting VEGF, SLIT3, and PDGF-BB. Additionally, type H vascular ECs secrete RANKL, which supports vessel-associated osteoclasts through a RANKL-RANK signalling mechanism, thereby facilitating bone regeneration. Based on (Zhang et al., 2020).

A particular vascular endothelial cell exists between osteoblasts and osteoclasts to promote angiogenesis, called H-type vascular endothelial cells, which can actively guide bone formation by producing inducible factors (Zhang et al., 2020). H-type blood vessels regulate the growth of skeletal vasculature and the function of osteoblasts, which are essential regulators of bone regeneration (Peng et al., 2020). H-type vascular endothelial cells are surrounded by osteoprogenitor cells expressing osteoblast-specific transcription factors (Wang et al., 2017). These include osteoprogenitor cells expressing Runt-related transcription factor (Runx2), osteogenesis-related transcription factor, and osteoblasts expressing type I collagen (Yang et al., 2017). Osterix promotes bone formation effectively, Runx2 is an essential transcription factor for osteoblast differentiation, and type I collagen is involved in the stages of osteocyte proliferation, differentiation, and mineralization. In addition, H-type vascular endothelial cells promote the survival, proliferation, and differentiation of bone progenitor cells by secreting growth factors, such as PDGF, TGF⁃β1, TGF⁃β3, and FGF⁃1, etc., (Zhao and Xie, 2020). Osteoprogenitor cells are close to H-type blood vessels, which provide osteoblast resources for osteogenesis, while oxygen offers the necessary nutrients to support osteogenesis’ metabolic demands. Therefore, H-type vascular endothelium regulates osteoblasts and osteoclasts and is an essential regulator of bone regeneration (Li et al., 2019). By combining signaling pathways and cytokines, H-type blood vessels play a vital role for bone formation and blood vessel growth. Figure 1 illustrates that the multiple signalling factors are involved in coupling of type H vessel formation and osteogenesis in the bone marrow microenvironment.

### 2.2 Osteogenic differentiation promotes angiogenesis

The H-type blood vessels is also regulated by a number of cell-derived cytokines. Various cytokines are secreted into the bone marrow microenvironment to induce endothelial cell proliferation, vascular assembly, and stabilization, providing sufficient guarantee for forming H-type blood vessels (Xu et al., 2018). HIF-1α, Notch, and VEGF can promote vascular assembly stabilization and bone formation (Zhu et al., 2020). VEGF is one of the most effective cytokines for inducing angiogenesis and is secreted by cells involved in bone development and repair, including endothelial cells, osteoblasts, and hypertrophic chondrocytes (Dreyer et al., 2020). VEGF mainly acts on endothelial cells, stimulates the formation of new blood vessels, and brings osteoprogenitor cells into the callus site to promote bone formation. Notch signaling is the key linking angiogenesis, vascular secretion signaling, and osteogenesis and is also a critical pathway for regulating the sprouting of new blood vessels in bone (AlMuraikhi et al., 2019; Guo et al., 2020). Under the condition of hypoxia or hypoxia, HIF-1α produced by osteoblasts promotes angiogenesis and the expression of VEGF-A (Hu and Olsen, 2016a). At the same time, an increase in HIF-1α expression in cells led to a significant expansion of H-type vascular endothelial cells, Osterix, and Runx2 osteoprogenitor cells (Hu and Olsen, 2016b). Slit homologous protein 3 (SLIT3) and PDGF-BB can bind to the corresponding endothelial cell receptors to further enhance VEGF secretion and stimulate blood vessel growth (Xu et al., 2018; Gao et al., 2019). In addition, miRNAs can also regulate H-type angiogenesis through various signaling pathways related to bone metabolism (Chen et al., 2020; Wang et al., 2020).

## 3 Effects of physicochemical properties of bone bioscaffolds on angiogenesis

Bone tissue engineering technology typically comprises three fundamental components: a biomaterial scaffold, bioactive factors, and graft cells. Among these, the biomaterial scaffold plays a central role in offering mechanical stability to the bone defect site, while also serving as a carrier for the bioactive factors and graft cells. The biomaterial scaffold is also crucial for achieving spatio-temporal regulation of the bioactive factors and effectively influencing the regeneration of blood vessels. The structural parameters and surface properties of bone grafts are closely related to cellular responses and angiogenesis and ultimately affect bone formation, including surface properties, pore shape, porosity, and fiber orientation (Bobbert and Zadpoor, 2017; Ortiz et al., 2018; Bank, 2019; Hayashi et al., 2020). Understanding the effect of bone substitute structure on angiogenesis is essential for optimizing the design of porous biomaterials targeting bone regeneration. By optimizing the parameters of biomimetic materials to enhance the synergistic interaction among scaffolding materials, bioactive factors, and cells within a biological milieu, these biomaterials facilitate the enhancement of bone regeneration and angiogenesis, thereby directing the process of new bone formation (Table 2).

**TABLE 2:** Effects of physical and chemical properties of materials on bone angiogenesis.

Type	Characteristic	Effect	Ref
Surface morphology	PFCH fibrous membranes with random, aligned and latticed topography	Recruit macrophage/monocyte and induce angiogenesis	Jin et al. (2021)
	Surface of biopolymer nanofibers with CNTs to create a unique bi-modal nanoscale topography (500 nm CNTs with 25 nm CNTs)	Reduce inflammation, promoting angiogenesis, and driving adhesion and osteogenesis of MSCs	Patel et al. (2020)
	Dexamethasone-loaded biphasic calcium phosphate nanoparticles/collagen composite scafolds with several types of concave microgrooves	facilitated angiogenesis and stimulated new bone formation	Chen et al. (2018)
	A sponge-like scaffold with hierarchical and interconnected pores	cell-material interaction promotion and MSC paracrine function modulation effects	Liu et al. (2020)
Porosity and pore size	A highly porous and elastic aerogel made from ultralong HAP nanowires with ultrahigh porosity (B98.5%), excellent elasticity and suitable porous structure	ingrowth of new bone and blood vessels	Huang et al. (2021)
	The architecture of larger pores at the periphery of graded scaffold	Enhance angiogenesis and osteogenesis	Yang et al. (2015)
Pore structure	Multi-level pores into the biocompatible scaffolds	promote angiogenesis and bone regeneration	Liu et al. (2020)
	Scaffolds with irregular pore sizes	be more conducive to bone tissue ingrowth and blood vessel formation	Wang et al. (2021)
Other	The 3R02 bivalent aptamer specific to VEGF was grafted to the HA surface	promotes bone regeneration and angiogenesis	Brydone et al. (2010)

PFCH., Poly (lactate-co-glycolate)/fish collagen/nano-hydroxyapatite.

CNTs. Carbon nanotubes.

HAP., hydroxyapatite.

### 3.1 Surface topography

The interaction between biomaterials and the host is crucial for successful regeneration during bone tissue engineering. The biomaterial’s surface interacts with the host tissue fluid and cells, directly determining its mechanical binding and biological response to the host tissue (Bank, 2019). Optimizing the surface properties of biomaterials is a promising strategy to control various responses of cells involved in tissue repair processes, such as cell migration, adhesion, and ECM secretion (Denchai et al., 2018; Ortiz et al., 2018; Conserva et al., 2019; Huang et al., 2019; Richbourg et al., 2019). By comparing three different surface microstructures of electrospinning fiber membrane effect on blood vessels and osteogenesis, Jin et al. found that the reticular structure of fiber membrane promotes angiogenesis, macrophage recruitment, and osteogenesis, indicating that the reticular formation of the fiber surface can be used to regenerate bone as a surface design biomaterial (Jin et al., 2021). Nanotopological surfaces have been shown to modulate cell behaviors, such as initial adhesion, spreading, and differentiation. The application of nanomaterials to tailor the surface of polymer scaffolds can regulate and enhance biological responses with cells and enable tissue repair processes. For example, carbon nanotubes (CNTs) are used to modify the surface of biopolymer nanofibers to create unique bimodal nanoscale topography (500 nm nanofibers and 25 nm nanotubes), which can reduce inflammation, promote angiogenesis, and drive the adhesion of MSCs. It effectively promotes tissue healing and bone regeneration (Patel et al., 2020). Additionally, microgrooves added to the surface of composite scaffolds also aided in angiogenesis and bone regeneration. The micro-grooved structure helps guide human umbilical vascular endothelial cells (HUVECs) assembly into well-arranged tubular structures, promoting rapid angiogenesis (Chen et al., 2018).

### 3.2 Porosity and pore size

Pores in bone tissue biomaterials allow bone tissue to grow and blood vessels to form, which plays an important role in bone regeneration. It is crucial that the implant has a functional vascular network that delivers oxygen, nutrients, and signaling molecules, as well as removes metabolic waste and carbon dioxide (Bobbert and Zadpoor, 2017). Angiogenesis is also closely related to the pore structure in the scaffold (Yilgor et al., 2013; Vezenkova and Locs, 2022) and ultimately affects bone formation. Relevant parameters of pore structure in the scaffold include pore size (Freitas et al., 2022), orientation (Yilgor et al., 2013), uniformity (Wang et al., 2014; Vissers et al., 2015), interconnectivity (Vissers et al., 2015), and porosity (Ahn et al., 2018; Dou et al., 2021). These parameters are interrelated and coordinately influence the effect of bone regeneration. The effective threshold of aperture for bone regeneration was 100 μm (Bobbert and Zadpoor, 2017). Generally, when the pore size is less than 100 μm, the cell distribution and angiogenesis of the whole scaffold are limited, cell viability is reduced, cell proliferation and differentiation are delayed, and fibrous tissue is formed within the pores instead of bone (Bobbert and Zadpoor, 2017). Therefore, most studies have developed scaffolds with pore sizes exceeding 100 μm. Research reports vary on the importance of macropore size thresholds: some claim that macropores need to be larger than 400 μm for new bone to develop (Sicchieri et al., 2012). The threshold for pore size is closely related to the porosity, pore geometry, uniformity, orientation, interconnectivity, and chemical composition of different materials (Hayashi et al., 2020). The high-porosity scaffold facilitates osteoblasts' adhesion, proliferation, and migration and promotes angiogenesis and new bone formation in the defect area (Wang et al., 2021). For example, ultralong hydroxyapatite nanowires are assembled into cancellous bone-like aerogels by freeze-drying method, which has ultra-high porosity and multi-scale pores, providing a good environment for cell adhesion, proliferation, migration, and bone growth, and promoting osteogenesis and angiogenesis both *in vitro* and *in vivo* (Huang et al., 2021).

### 3.3 Pore structure

Evidence shows graded porous scaffolds are more effective at regenerating bone than scaffolds with uniform pore sizes (Yang et al., 2015; Liu et al., 2020). These gradient structures can promote cell migration, spreading, signaling, proliferation, and differentiation. Yang et al. fabricated a gradient porous scaffold (large central pores between 600 and 800 μm, tiny peripheral pores between 350 and 500 μm) and a scaffold with uniform pore sizes by template casting (Yang et al., 2015). The volume of newly formed bone in the gradient porous scaffold was significantly more prominent than in the scaffold with uniform pores *in vivo*. A gradient porous structure, which promotes angiogenesis and bone regeneration, is therefore a promising approach. The gradient porous scaffolds with large central and peripheral tiny pores can encourage tissue bone formation more than the uniform pore scaffolds. However, the macroporous structure around the gradient scaffold can promote angiogenesis and osteogenesis more than the small pore structure around the gradient scaffold (Li et al., 2020a). In addition, a 3D sponge scaffold with layered and interconnected pores promotes cell-material interactions, regulates the paracrine function of MSCs, and significantly supports vascularized bone regeneration (Lian et al., 2021).

### 3.4 3D printing promotes vascularized osteogenesis

3D bioprinting is a technology that combines 3D printing, tissue engineering, developmental biology and regenerative medicine to build bionic tissues (Vijayavenkataraman et al., 2018). 3D bioprinting allows spatially controlled and layer-by-layer deposition of specific biomaterials, cells and biomolecules to create complex vascularized tissues and intricate 3D structures (Pati and Cho, 2017). Compared with traditional methods, 3D bioprinting technology, which allows precise control of complex three-dimensional structures, multiple compositions, and spatial distributions (Vijayavenkataraman et al., 2018), has unparalleled advantages and is gradually gaining widespread attention. Kerouredan et al. (Kerouredan et al., 2019a) precisely printed high-density endothelial cells onto hydrogels containing mesenchymal stem cells to generate microvascular networks with specific structures. Piard et al. (Piard et al., 2019) developed an innovative fibrin-polycaprolactone composite scaffold, incorporating HUVECs, BMSCs, and fibronectin, through the utilization of extrusion 3D bioprinting. The resulting scaffold exhibited mechanical properties akin to bone, and subsequent *in vitro* and *in vivo* investigations demonstrated its commendable vascularization potential. Anada et al. (Anada et al., 2019) employed digital light processing to fabricate a dual 3D hydrogel structure, comprising an outer ring of gelatin methacrylate embedded with octacalcium phosphate and an inner ring of gelatin methacrylate containing HUVECs. This construct successfully facilitated the differentiation of BMSCs into osteoblasts and promoted the formation of blood vessels, thereby mimicking the process of bone formation in the human body. To simulate the vascularization of bone tissue, Shahabipour et al. (Shahabipour et al., 2022) used coaxial microextrusion to load HUVECs and osteoblasts (MC3T3) into the core and shell of bioink containing angiogenic and osteogenic factors, respectively, to form core-shell structures and indirect co-culture, and the scaffolds prepared demonstrated that the coaxial bioprinting technology and the indirect co-culture system can be used to construct bioreactive tissues with higher efficiency.

The angiogenesis of larger implants may be too slow, resulting in cell death or loss of function in the central region of the implant. To address this issue, prefabricated scaffolds or engineered tissues with a capillary-like network prior to implantation is a viable approach (Sharma et al., 2019). Such engineered vascular networks have been shown to anastomose with the host vascular system after implantation (Zhu et al., 2017). Kerouredan et al. (Kerouredan et al., 2019b) used laser-assisted bioprinting to implant labeled endothelial cells and collagen containing BMSCs and VEGF into cranial bone defects in mice to promote bone regeneration *in vivo* and to increase angiogenesis in bone defects. Nulty et al. (Nulty et al., 2021) co-cultured HUVECs, hBMSCs cells and 3D bioprinted them with different bioinks. *In vitro* 3D bioprinting of pre-designed sizes and shapes of implants and applying them to bone defects demonstrated that pre-vascularized bone tissues enhanced blood vessel formation in critical bone defects.

### 3.5 Microfluidics promotes vascularized osteogenesis

Microfluidics is a technology that precisely manipulates fluids in the micron scale, and the volume of the manipulated fluid can be as small as 10^−9^ ∼ 10^−18^ L (Long et al., 2009). Injectable fillers have been found to be applicable in minimally invasive procedures and are also deemed suitable for bone defects with intricate shapes (Brydone et al., 2010). Moreover, injectable fillers can serve as drug delivery systems and, owing to their resemblance to the extracellular matrix (ECM), they can facilitate the proliferation and adhesion of MSCs while acting as osteogenic supports (Liu et al., 2017; Yousefi, 2019). Hydrogel microspheres, as a representative material of injectable fillers, have been widely studied in bone tissue engineering and tissue regeneration (Li et al., 2021; Sun et al., 2022). Cheng et al. (Cheng et al., 2023) prepared nanohydroxyapatite/chitosan (nHA/CS) composite hydrogel microspheres with uniform particle size distribution by microfluidic technology. The composite hydrogel was demonstrated by *in vivo* and thus *in vitro* experiments to promote osteogenic differentiation of mesenchymal stem cells and vascularization of endothelial cells by controlling the release of strontium ions, thus accelerating the repair of bone defects. Yang et al. (Yang et al., 2023) developed VEGF-loaded pearl powder hybrid hydrogel scaffolds for bone regeneration using microfluidics combined with 3D printing technology. In this process, microfluidics combined with 3D printing technology can precisely shape the scaffolds for different application purposes. The composite hydrogel promotes revascularization of the damaged area by loading and releasing VEGF, which improves the delivery of oxygen and nutrients in the early stage of bone repair.

## 4 The effect of cytokine-loaded bone bioscaffolds on angiogenesis

Growth factors directly or indirectly regulate endothelial cell migration, proliferation, and aggregation, so they play a crucial role in the formation of vascularization (Sun et al., 2021). Currently, known angiogenic growth factors can be divided into the following categories. 1) Only endothelial cells are targeted cells that promote angiogenesis and generation, including VEGF and angiopoietin. The former acts on the early stage of vascularization and promotes the formation of the original vascular network. The latter acts on the subsequent vascular remodeling and shaping and promotes the maturation of the vascular network. 2) It acts on various endothelial cells and directly affects vascularization, including growth factors and chemokines. A typical representative is FGF, which can promote endothelial cell division and chemotactic endothelial cells and strongly promotes angiogenesis. 3) It indirectly affects vascularization, mainly inducing the release of the above two types of factors to stimulate angiogenesis, including TGF-β and platelet-derived factors. The primary function of the former is to maintain the integrity of the vascular wall. At the same time, the latter promotes the division of vascular smooth muscle cells to promote vascular maturation and maintain vascular stability. Table 3 shows several cytokines loaded with bone biomaterials that promote angiogenesis and osteogenesis. Among all these factors, VEGF is the most commonly used in bone tissue engineering (Li et al., 2020b; Kauffmann et al., 2021).

**TABLE 3:** Effect of cytokine-loaded materials on bone angiogenesis.

Factor	Carrier	Effect	Ref
VEGF	Polydopamine-Coated Poly (l-lactide) Nanofibers	Promote angiogenesis and osteogenesis	Li et al. (2020a)
	Hierarchical micro/nanofibrous membranes	A sustained release	Wu et al. (2020)
		Mimicked the microenvironment of extracellular matrix	
	Chemotactic Functional Scaffold	Stimulate mobilization and homing of MSCs to sites of bone loss	Brydone et al. (2010)
		A novel nucleotide aptamer-functionalized fibrin hydrogel (AFH)	
		Aptamer functionalization improve the release kinetics of VEGF from AFH.	
	Microbubbles	Combination with ultrasound-targeted microbubble destruction (UTMD)	Sharma et al. (2019)
Deferoxamine	Poly (lactic-co-glycolic acid)	Activate the HIF-1a signaling pathway stimulating production of VEGF and other downstream angiogenic factors	Kerouredan et al. (2019b)
	3D bioglass-nanoclay scaffolds	Sustained DFO release and inhibited DFO degradation	Zheng et al. (2021)
	Hydrogel	Multiple osteoinduction cues including organic–inorganic compositions, and angiogenic/osteoinductive factors	Pati and Cho (2017)
Angiopoietin 2	A hydroxyapatite/collagen scaffold	Promoted angiogenesis and improved repair of bone defects by inducing autophagy	Yin et al. (2018)

PA., peptide amphiphile.

SNF., silk nanofibers.

HA., hydroxyapatite nanoparticles.

There are mainly two different ways for growth factors to be applied in tissue engineering bone construction: one is to compound growth factors directly on the scaffold or to combine with it after the scaffold is constructed (Li et al., 2016; Yin et al., 2018; Cheng et al., 2020; Zheng et al., 2021). The other is to simultaneously transplant cells that can secrete growth factors on the scaffold. These cells can secrete growth factors in the natural state (Kang et al., 2014) or be modified by genetic engineering (Li et al., 2016; Li et al., 2017). However, as a dynamic and comprehensive process, bone regeneration requires the precise release of a certain dose of growth factors and signaling molecules at specific stages. Therefore, the simulation is not only about releasing growth factors from the drug delivery or presentation system *in vivo*. Since drugs' rapid or continuous release cannot ensure accurate coordination with the dynamic environment *in vivo*, it is essential to coordinate multi-dimensional factors such as time and dose in bone tissue regeneration and repair strategies.

Since growth factors lack stability and are readily inactivated, they decompose too quickly to be effective alone. Most reports use growth factors in combination with appropriate scaffold materials at specific target sites to achieve effective delivery and controllable release of growth factors (Jia et al., 2016; Wu et al., 2020). For example, a burst-release microbubble system loaded with VEGF can be developed to achieve the directional and concentrated release of VEGF through targeted microbubble destruction by ultrasound, which can significantly enhance the growth of blood vessels and new bone formation in bone defects (Gong et al., 2019). At present, the therapeutic methods of angiogenesis emphasize the local concentration and continuous use of growth factors rather than local and single rapid intravenous injection, and many natural, synthetic, and composite materials have been used as the release vectors of angiogenic growth factors. For example, some scholars have developed a sequential growth factor slow-release double-freezing hydrogel system. The outer layer is gelatin/chitosan frozen gel wrapped with VEGF, and the inner layer is gelatin/heparin frozen gel containing bone morphogenetic protein-4 (BMP-4). It promotes angiogenesis and osteogenesis through early release of VEGF and sustained release of BMP-4 (Lee et al., 2020). Of course, grafting the 3R02 bivalent nucleic acid aptamer specific to VEGF is also a good choice onto the surface of hydroxyapatite (HA). This aptamer-conjugated hydroxyapatite (Apt-HA) shows higher VEGF protein capture ability and faster growth of HUVEC (Son et al., 2019).

In addition, VEGF and various cytokines can exert synergistic effects and play a vital role in the interaction between osteoblasts and endothelial cells (Dohle et al., 2018; Simunovic et al., 2019). Wang et al. introduced BMP-2 and VEGF into silk fibroin microspheres and then incorporated these microspheres into nano-hydroxyapatite scaffolds to provide controlled release. The results showed that this scaffold’s sustained-release extremely low-dose BMP-2 and VEGF synergistically affected vascularized bone regeneration. Compared with other systems, this controlled release system can significantly reduce the use of BMP-2 (Wang et al., 2017). The chemotactic effect of VEGF in angiogenesis was utilized to promote the recruitment and differentiation of osteoprogenitor cells into osteoblasts by activating specific receptors and to guide the migration of MSCs to bone defects. Based on this, some scholars have used amphiphilic polypeptide (PA) nanofiber gel to wrap vascular endothelial growth factor to form a chemotactic functional scaffold. It can guide stem cells to migrate to the defect area and induce differentiation by controlling the release of VEGF to promote angiogenesis and osteogenesis (Bakshi et al., 2021).

Although the engineering biomaterials combined with growth factors have achieved good results in inducing bone repair and angiogenesis, the clinical application of growth factors is limited by their short half-life, easy inactivation, and therapeutic effect exceeding the physiological dose. The transmission of growth factor genes rather than simple growth factors has been proven to be an effective way to maintain biological activity to treat bone defects and diseases (Chen et al., 2011). Viral vector has become a promising transmission tool for tissue regeneration because of its high expression efficiency. The gene fragment with growth factor is transfected into seed cells by vector virus; seed cells can express the growth factor stably and continuously. After implantation at the target location, the cell will have a more vital ability to promote vascularization. Releasing the transfection vector that can express the growth factor gene from the scaffold effectively improves bone regeneration. For this purpose, viral vectors are commonly used due to their high efficiency and stability in delivering genes (Chang et al., 2009).

With drug-controlled release and non-viral vector technology development, Superparamagnetic gene microspheres with biodegradable and controlled gene release have emerged as the most promising carriers for cellular functions in scaffolds. This can generate revolutionary innovation in bone tissue engineering research. A non-invasive oscillating and static magnetic field was used to drive the micromotion of magnetic gene-loaded microspheres in the bone biomaterials to promote the release of plasmid genes. It has been shown that plasmids are released and enriched locally in VEGF magnetic microspheres under oscillating and static magnetic fields. It was also observed to promote vascularization and osteogenesis of artificial bone *in vivo* (Luo et al., 2018).

## 5 Effect of trace elements on angiogenesis of bone biological scaffold

Bones comprise about 20% collagen and 70% minerals, while other organic materials such as proteins, polysaccharides, and lipids account for only a tiny portion of bones (Lin et al., 2019). Bone minerals contain many trace elements, such as magnesium, calcium, zinc, and strontium, which are involved in many processes related to bone regeneration (Kristina et al., 2018). Therefore, combining these natural bioactive ions with scaffolds can provide a safer alternative strategy for bone regeneration (Table 4). Recently, it has been reported that ions such as magnesium, silicon, and copper can stimulate angiogenesis and promote new bone formation (Lai et al., 2019). Compared with growth factors, incorporating bioactive ions into bone substitutes is a simpler and safer method to encourage bone regeneration, and the cost is relatively low.

**TABLE 4:** Effect of materials loaded with trace elements on bone angiogenesis.

Metal	Carrier	Effect	Ref
Mg	3D printed porous tantalum scaffolds	Improve surface bioactivity and lead to better osteogenesis and angiogenesis	Lee et al. (2020)
	3D printed Mg-doped TCP scaffolds	Alter physicochemical properties of cells, enhance the osteogenesis and angiogenesis of bioceramic scaffolds	Sun et al. (2021a)
	Dual-crosslinked hydrogel	Promote cell adhesion, osteogenesis and vascularization	Li et al. (2020b)
	Microfluidic Hydrogel Microspheres	Capture Magnesium, sustained release performance, bone-targeting properties	Li et al. (2017)
strontium	Hybrid cement	Improve mechanical strength and handling properties, improve both biological and physicochemical properties	Son et al. (2019)
	Microspheres	Biomineralized, injectable and biodegradable	Bakshi et al. (2021)
		Induce bone regeneration and kill bacteria	
	3D printed porous scaffolds	Manipulates favorable macrophages activation to facilitate osteogenesis/angiogenesis	Wang et al. (2017b)
	Ceramics	Release of exosomal miR-146a, promote osteogenesis and angiogenesis	de Laia et al. (2021)
Cobalt	Bioceramics	Appropriate Co promote osteogenesis, excessive Co suppressed bone formation	Kristina et al. (2018)
	Metal−Organic Framework Hydrogel Nanocomposites	A powerful proangiogenic/osteogenic agent, tunable and Controlled Release of Cobalt Ions	Lai et al. (2019)
	Bioceramic scafolds	Enhance osteogenic and angiogenic properties	Nabiyouni et al. (2018)
Copper	Bioactive glass composite scaffolds	Activate the HIF-1α and TNF-α pathway of hUVECs	Nabiyouni et al. (2018)
	Mesoporous bioactive glass	Accelerated host angiogenesis and immune responses	Ma et al. (2020)

### 5.1 Magnesium

A vital component of bone development and maturation, magnesium occupies the second most abundant position in natural bones after calcium (Gu et al., 2019). Magnesium ion regulates many enzymes and cell functions and significantly impacts cell homeostasis (Lai et al., 2019). In addition, magnesium ions can improve cell adhesion and differentiation (Zhang et al., 2021) and regulate local bone formation by promoting angiogenesis (Yu et al., 2017).

Several attempts have been made in recent years to stimulate vascularized bone repair by adding bioactive magnesium ions to bone substitutes. For example, a magnesium-rich microsphere was made to simulate the bone microenvironment, thus inducing vascularized bone formation (Lin et al., 2019). Yu et al. reported a composite carrier containing Mg2+, which showed excellent angiogenic ability. Zhang et al. developed a double-crosslinked hydrogel containing magnesium ions to repair bone defects by promoting cell adhesion, angiogenesis, and osteogenesis (Zhang et al., 2021). Furthermore, magnesium surface biological coatings and magnesium-based bone cement also effectively promote angiogenesis (Nabiyouni et al., 2018; Ma et al., 2020).

There is, however, a problem with most magnesium-based bone biomaterials due to their passive mixing with metal magnesium, which has a defect of fast release rate. The rapid release will cause excessive magnesium hydroxide, forming a “high magnesium microenvironment” around the implant, destroying calcium-related physiological processes in surrounding cells and tissues and even causing toxic damage (Kuśnierczyk and Basista, 2016). Inspired by the attraction of metals by magnets, Zhao et al. developed bisphosphonate functionalized injectable hydrogel microspheres by using the coordination reaction of metal ion ligands, which can promote cancellous bone reconstruction in osteoporotic bone defects by capturing Mg^2+^. The experimental results show that Mg^2+^ trapping composite microspheres excited by magnets can stimulate the function of osteoblasts and endothelial cells' function and inhibit osteoclasts’ activation, which is beneficial to angiogenesis and osteogenesis and effectively promotes bone regeneration (Zhao et al., 2021).

### 5.2 Strontium

Strontium (sr) is an essential trace element in the human body and a regular component of bones. Many studies have shown that strontium can promote osteoblast generation, stimulate bone development and growth, inhibit the process of bone resorption, and maintain the normal physiological function of the human body (Pilmane et al., 2017). Strontium ranelate (SrR) has been proven effective in treating osteoporosis in postmenopausal women (Lin et al., 2021). In addition, Sr or SrR has also been shown to promote angiogenesis (Bakshi et al., 2021) effectively. For example, SrR can promote angiogenesis and differentiation of OVX-BMSCs and HUVECs, increase the mRNA levels of VEGF and Ang-1 in osteoporotic BMSCs, and increase the mRNA level of VEGF in HUVECs (Guo et al., 2016).

There is great potential in treating bone defects with strontium-doped bone tissue biomaterials with dual osteogenesis and angiogenesis effects. For example, to improve the physicochemical and biological properties of calcium phosphate mixed cement (CPHC), a strontium-reinforced CPC (Sr-CPHC) was developed. It was found that Sr-CPHC significantly promoted the endothelial cell migration and tube formation of HUVECs *in vitro* and upregulated the expression of VEGF and angiopoietin-1 (Ang-1) (Wu et al., 2021). Experiments *in vivo* and *in vitro* showed good osteogenic function.

In addition, the design of new composite bone biomaterials aimed at combining the complementary biological effects of various beneficial elements has also achieved good results (Baba et al., 2017; Ratnayake et al., 2017). For example, Sr2+/Fe3+ co-substituted nano-hydroxyapatite directly promoted the function of MC3T3 osteoblasts and HUVECs and greatly facilitated the activation of M2 macrophages to promote osteogenesis and angiogenesis. The results of subcutaneous implantation *in vivo* and repair of skull defects further confirmed the excellent ability of Sr2+/Fe3+ in immune regulation, angiogenesis, and bone regeneration *in situ* (Yang et al., 2021).

### 5.3 Cobalt

Cobalt (Co) plays a crucial role in the human body, which protects HIF-1a from degradation by inhibiting proline hydroxylase (PHD), simulates hypoxia, activates HIF-1a signal pathway, and then upregulates the expression of targeted genes to promote angiogenesis (Pacary et al., 2006). In addition, cobalt ions can modulate the expression of nuclear factor κB (NFkB), triggering the transcriptional activation of HIF-1α (Kulanthaivel et al., 2016). HIF-1 α can upregulate the expression of angiogenic genes (VEGF, bFGF, and SDF-1) related to bone regeneration (Hu et al., 2008; Zou et al., 2012).

Cobalt’s angiogenic properties are widely used in tissue engineering, such as cobalt-containing bioactive glass stimulates angiogenesis and gene expression of HIF-1 α and VEGF (de Laia et al., 2021). The vascularization of the bone is vital for the repair of defects. Insufficient or abnormal vascularization can lead to cell death or poor bone formation due to deficient nutrient and oxygen supply. The dose effect of vascularization on bone formation was studied by tricalcium phosphate (TCP) scaffolds doped with different concentrations of cobalt. The study also found that TCP doped with Co positively influenced osteogenesis, but excessive Co inhibited differentiation of osteoblasts and bone formation (Zheng et al., 2019).

There is excellent potential for bone repair with cobalt ions as they simulate hypoxia to stimulate angiogenesis (Li et al., 2021). However, the release curve of cobalt ions is the key to better use of cobalt ions to repair bone defects. Sun et al. prepared in-situ photocrosslinked nanocomposite hydrogel as a controlled release system for regulating Co ions, which can maintain the continuous release of Co ions for 21 days, which matches the early angiogenesis in the process of bone formation. Experiments *In vivo* and *in vitro* showed good ability for bone formation and new blood vessel formation (Sun et al., 2021).

### 5.4 Copper

In the body, copper (Cu) is an essential micronutrient that participates in many biological processes and maintains many enzymes' homeostasis and physiological function (Shi et al., 2016; Dong et al., 2020). Cu can stimulate human endothelial cell proliferation and VEGF secretion, thus promoting angiogenesis (Rau et al., 2019). Copper ion is closely related to fibroblast growth factor, and they synergistically stimulate angiogenesis. VEGF activates endothelial cells by regulating the proliferation and migration of endothelial cells in surrounding tissues and finally forming tubular structures. Copper ions can also promote endothelial cells' recruitment, differentiation, and angiogenesis by increasing the expression of HIF 1α and inhibiting the degradation of hypoxia-inducible factor 1 α (Magri et al., 2017). At the same time, the activation of HIF-1α can promote the transcription of VEGF expression.

The characteristics of copper ion angiogenesis to promote bone repair are widely used in bone biomaterial engineering. In one study, copper-added Cu-CPS ceramics [Cu-Ca5 (PO4) 2SiO4] showed better angiogenic and osteogenic properties than pure CPS ceramics. It was found that Cu played an essential role in promoting angiogenesis in the early stage, and the synergistic effect of silicon and copper promoted osteogenesis and angiogenesis in the later stage (Wu et al., 2021). In addition, adding Cu can enhance the biological activity of cells in the bioactive glass field because Cu has osteogenic and angiogenic properties (Zhou et al., 2018; Dai et al., 2021).

### 5.5 Silicon

As one of the necessary trace elements for the growth and development of organisms, silicon can promote angiogenesis and the formation of functional vascular networks, participate in the regulation of bone formation and calcification, and play an essential role in bone metabolism (Khandmaa et al., 2017a; Yan et al., 2018). Silicon ions promote angiogenesis by inhibiting the expression of prolyl hydroxylase-2 and upregulating the HIF-1 α signal pathway (Khandmaa et al., 2017b).

It has been found that doping silicon-coated implants can promote angiogenesis by promoting adhesion, migration, proliferation, tube formation, and angiogenesis-related gene expression of HUVEC (Fu et al., 2020; Monte et al., 2020). Cell-cell interaction can promote silicon-mediated angiogenesis through a paracrine pathway. In one study, it was found that silicon ions did not promote the expression of the VEGF gene in individual cultured human dermal fibroblasts (human dermal fibroblast, HDF) and did not upregulate the expression of vascular endothelial cadherin (VE-cad) cad in cultured HUVEC cells alone. However, silicon ions greatly enhanced the expression of the VEGF gene in HDF cells co-cultured with HDF and HUVEC and then upregulated the expression of the KDR receptor in the co-cultured HUVEC through the paracrine pathway, which activate the expression of VEGF/KDR/eNOS/NO axis and VE-cad gene to promote functional angiogenesis (Li and Chang, 2013). In addition, It was found that strontium-substituted calcium silicate ceramics can stimulate BMSCs to release exosomal miR-146a to regulate osteogenesis and angiogenesis (Liu et al., 2021).

## 6 Conclusion

In conclusion, there is a close relationship between blood vessels and bone in bone growth and development and maintaining bone mass balance. The angiogenic-osteogenic coupling involves many growth factors, cells, and regulatory effects. The success of bone regeneration induced by biomaterials depends on the early promotion of angiogenesis and the regulation of critical links in bone healing. By optimizing the scaffold material and improving the parameters of the scaffold material, a scaffold with a specific structure suitable for vascular growth can indeed be made. However, the scaffold with optimized structure still needs vascular growth of the body after implantation, so it is limited to increasing the vascularization of tissue engineering bone only by optimizing the scaffold material. The effect of promoting vascular growth factor is positive, but this method still has difficulties in growth factor dose control and sustained release technology. Insufficient local release concentration and short duration can not achieve a good effect of inducing regeneration. Too high a release concentration can even produce cytotoxicity. Gene transfection technology no longer has the problem of controlled release of growth factors. Still, the characteristics and function of seed cells transfected with genes may be affected and face the risk of carcinogenesis. Trace elements and biomaterials were combined to modify bone repair materials to make them have good angiogenic properties. Trace elements can quickly pass through the cell membrane, regulate multiple cell physiological processes, improve the osteogenic microenvironment, and promote angiogenesis. However, most experiments lack research on the related mechanism, and relatively high concentrations of metal ions may have non-specific side effects on multiple systems and organs. Therefore, determining the optimal concentration of trace elements to promote angiogenesis and osteogenesis, controlling their release from scaffold materials, and elucidating their release kinetic basis *in vivo* and related immune regulatory mechanisms may be the issues that need to be addressed by future researchers.
